# Editorial: Emotionally intelligent leadership in medicine

**DOI:** 10.3389/fpsyg.2022.999184

**Published:** 2022-09-06

**Authors:** Bobbie Ann Adair White, Philip A. Cola, Richard Eleftherios Boyatzis, Joann Farrell Quinn

**Affiliations:** ^1^Department of Health Professions Education, School of Healthcare Leadership, MGH Institute of Health Professions, Boston, MA, United States; ^2^Case Western Reserve University, Cleveland, OH, United States; ^3^Department of Medical Education, University of South Florida, Tampa, FL, United States

**Keywords:** emotional intelligence (EI), medicine, social competencies, emotional competencies, leadership, nursing, medical education

## Introduction

Emotional intelligence (EI) is a Research Topic of increasing interest over the past couple of decades due to growing evidence that EI can be learned and is a key component of quality leadership (Boyatzis and McKee, 2005). For example, we have known for some time that EI competencies improve performance and work organizational outcomes (Sy et al., 2006; Boyatzis, 2018) and we know that these ideas are generalizable across the world culturally (Emmerling and Boyatzis, 2012; Meisler and Vigoda-Gadot, 2014) and disciplines (McCallin and Bamford, 2007; Boyatzis et al., 2017). While there is growing interest in medicine, there remains limited evidence on individual and organizational outcomes. The authors believe that the lack of progress in medicine can be attributed to a bias around the concept of EI within the medical community. We can point to numerous examples of physicians clinicians misunderstanding the social science of EI as “too touchy feely” for medical practitioners. Thus, for a community that is heavily rooted in hard sciences and known for their concreate thinking, it can be difficult to wrap their minds around something that feels ambiguous or abstract. To illustrate this point, we share that our work has often used EI as a “covertcurriculum,” hiding the underlying EI framework from the titles. Additionally, we have been advised to remove EI from our work to make it stronger or less likely to be dismissed (Quinn and White, 2019; Boyatzis and Feddeck, unpublished^1^). With these things in mind, we sought to continue to normalize and advance the use of EI within medicine and medical education through an article series entitled “*Emotionally Intelligent Leadership in Medicine*.” The hope was to bring the Research Topic to the forefront in the medical community to further advance EI in medicine.

This Research Topic of articles explores emotional and social competencies through a variety of frameworks, with a focus on a mixture of roles within different contexts and environments. From undergraduate medical education (UME), professional practice, surgery training and nursing, competencies are explored with outcomes related to leadership development, emotional resiliency, and behavioral change.

## Finding an emotional intelligence baseline in medicine

Petrides et al. found surgeons were equal in EI scores across surgical specialties, not finding a difference between specialties as previously hypothesized. Additionally, they found scores from surgeons to be higher than only one of their comparison groups, while exhibiting lower scores than the remaining four groups (executives, senior managers, salespeople, and nurses). Also of interest are the two areas that surgeons may need direct intervention; job satisfaction and job performance both of which are connected to wellbeing. Knowing that EI can be taught or remediated indicates this group could benefit from coaching or training in this space. “Coaching has the power to lead physicians and nurses to excellence (Deiorio et al., 2016) and is instrumental in reducing physician burnout (Dyrbye et al., 2019)” (as cited in Boyatzis and Feddeck, unpublished). We have noted previously that EI is critical to the development of physician and leadership identity in Medicine (Quinn and Perelli, 2016; Quinn and Cola, 2020).

Pelfrey et al. recognized the importance of coaching in accelerating the emotional and social development of women leaders in medicine. Through their leadership program, they were able to help women leaders in the following areas: communication skills, self-efficacy, networking, relationship capacity, situational awareness, and visioning, each of which are components of EI. Development of these individuals has led to advocacy and visibility, both of which are necessary to reduce the under-representation of women in academic leadership positions. Additional quantitative research from this group is in the works around the sustainability of EI competencies including self-efficacy. It is hoped that our series of articles here provided a launching point for even more work in EI competencies for medical professionals.

To establish additional baseline information, Pérez-Díaz et al. evaluated the trait EI construct across populations and sociodemographic variables. Their investigation established cross-cultural stability and validity, and Jiang et al. found EI plays a role in a nurse's ability to take advantage of resources, impacts job climate, and EI improved nurses' engagement scores.

## Emotional and social competencies

Grunberg et al. approached the Research Topic from a theoretical perspective, with a focus on social comparison theory and field theory as the basis for developing effective healthcare leaders through their own awareness; as well as regulation of their own and others' behaviors, cognitions, motivations, and emotions to become emotionally intelligent healthcare leaders.

Fernández-Rodríguez et al. and White et al. took an educational intervention approach to emotional and social competencies to improve empathy and emotional intelligence in medical students. Declining empathy in medical education has been of interest for a decade and Fernández-Rodríguez et al. brought forth a potential solution with their longitudinal study about medical semiotics training, which had a positive effect on empathy, moving this competency in the right direction. White et al. also sought to improve emotional intelligence through an educational intervention for their military medical students. Their research team found success in using a realistic surgical training course including mass-casualty scenarios to build students' hardiness and emotional intelligence.

## Suggestions and plans for future research

Petrides et al. further surfaced a need for longitudinal data in the space of medical training; this would be a great next step. Current literature demonstrates a decrease in empathy throughout medical education training (Neumann et al., 2011). Empathy is a component of EI, therefore, we can hypothesize that EI in the area of empathy decreases over time. There is evidence to show that surgeons score highly on scales like self-regard, stress tolerance, and optimism, while scoring less high on empathy, social responsibility, and impulse control (Stanton et al., 2011). Additionally, our contributors have shown that empathy and EI can be improved through educational interventions and coaching. Therefore, a large multi-center study looking at building emotional intelligence in medical leaders through coaching and education may be the next big step. More specifically, we would want to use coaching with compassion for building emotionally intelligent medical leaders and organizations (Boyatzis et al., 2019).

Finally, incorporating EI in Medicine is more critical than ever following increased rates of burnout and decreased performance post the COVID-19 pandemic. We are privileged and fortunate to be on the leading edge of providing a space where the intersections of medicine, management, and coaching can come together to improve leadership, training, education, performance, and outcomes in the practice of Medicine globally.

## Author contributions

JQ, BW, and PC drafted, revised, and edited the manuscript. RB provided final review and revisions of the manuscript. All authors contributed to the article and approved the submitted version.

## Conflict of interest

The authors declare that the research was conducted in the absence of any commercial or financial relationships that could be construed as a potential conflict of interest.

## Publisher's note

All claims expressed in this article are solely those of the authors and do not necessarily represent those of their affiliated organizations, or those of the publisher, the editors and the reviewers. Any product that may be evaluated in this article, or claim that may be made by its manufacturer, is not guaranteed or endorsed by the publisher.

## Footnotes

^1^Boyatzis, R. E., and Feddeck, M. V. (unpublished). Why Healthcare Needs Coaching with Compassion to Increase Emotional Intelligence at Four Levels. Cleveland, OH: Case Western Reserve University.

## References

[B1] Boyatzis R. Rochford K. Cavanagh K. V. (2017). Emotional intelligence competencies in engineer's effectiveness and engagement. Career Dev. Int. 2016, 136. 10.1108/CDI-08-2016-0136

[B2] Boyatzis R. E. (2018). The behavioral level of emotional intelligence and its measurement. Front. Psychol. 9, 1438. 10.3389/fpsyg.2018.01438 30150958 PMC6099111

[B4] Boyatzis R. E. McKee A. (2005). Resonant Leadership: Renewing Yourself and Connecting With Others Through Mindfulness, Hope, and Compassion. Boston, MA: Harvard Business School Press.

[B5] Boyatzis R. E. Smith M. Van Oosten E. (2019). Helping People Change: Coaching with Compassion for Lifelong Learning and Growth. Boston, MA: Harvard Business School Press.

[B6] Deiorio N. M. Carney P. A. Kahl L. E. Bonura E. M. Juve A. M. (2016). Coaching: a new model for academic and career achievement. Medical Educ. Onl. 21, 33480. 10.3402/meo.v21.33480 27914193 PMC5136126

[B7] Dyrbye L. N. Shanafelt T. D. Gill P. R. Satele D. V. West C. P. (2019). Effect of a professional coaching intervention on the well-being and distress of physicians. J. Am. Med. Assoc. Internal Med. 179, 1406. 10.1001/jamainternmed.2019.2425 31380892 PMC6686971

[B8] Emmerling R. J. Boyatzis R. E. (2012). Emotional and social intelligence competencies: cross cultural implications. Cross Cult. Manag. 19, 4–18. 10.1108/13527601211195592

[B9] McCallin A. Bamford A. (2007). Interdisciplinary teamwork: is the influence of emotional intelligence fully appreciated? J. Nurs. Manag. 15, 386–391. 10.1111/j.1365-2834.2007.00711.x 17456167

[B10] Meisler G. Vigoda-Gadot E. (2014). Perceived organizational politics, emotional intelligence and work outcomes: empirical exploration of direct and indirect effects. Personnel Rev. 43, 116–135. 10.1108/PR-02-2012-0040

[B11] Neumann M. Edelhäuser F. Tauschel D. Fischer M. R. Wirtz M. Woopen C.. (2011). Empathy decline and its reasons: a systematic review of studies with medical students and residents. Acad. Med. 86, 996–1009. 10.1097/ACM.0b013e318221e615 21670661

[B12] Quinn J. F. Cola P. (2020). Understanding physician leadership: the mediating effects of positive organizational climate and relational role endorsement. J. Bus. Indus. Market. 35, 1491–1503. 10.1108/JBIM-01-2019-0032

[B13] Quinn J. F. Perelli S. (2016). First and foremost, physicians: the clinical versus leadership identities of physician leaders. J. Health Org. Manag. 2015, 79. 10.1108/JHOM-05-2015-0079 27296888

[B14] Quinn J. F. White B. A. (2019). Cultivating Leadership in Medicine. Dubuque, IA: Kendall Hunt.

[B15] Stanton C. Sethi F. Dale O. Phelan M. Laban J. Eliahoo J. (2011). Comparison of emotional intelligence between psychiatrists and surgeons. Psychiatrist. 35, 124–129. 10.1192/pb.bp.110.029959

[B16] Sy T. Tram S. O'hara L. A. (2006). Relation of employee and manager emotional intelligence to job satisfaction and performance. J. Voc. Behav. 68, 461–473. 10.1016/j.jvb.2005.10.003

